# Fulton County Medical Society, May 16th, 1907—Headache

**Published:** 1907-07

**Authors:** Wesley Taylor

**Affiliations:** Atlanta, Ga.


### FULTON COUNTY MEDICAL SOCIETY, MAY 16th, 1907.

---

### HEADACHE.*

---

Dr. Wesley Taylor took up headache as a symptom and not as a disease, and separated it from neuralgia and migraine. As to the pathology he did not undertake to say what it is that aches. Experiments tend to show that it is not the medullary substance that aches, but rather the basal ganglia. Tumors may even exist in the substance and not cause any painful symptoms. He called attention to a case under his own observation where a tumor had existed until death without causing pain and had never been suspected 'till it was accidentally found at post-mortem. Attention is called to

*Author's Abstract.

the fact that in the case of idiots and imbeciles and even in hemiplegic children, headache is a really rare occurrence, which goes to show that when the cortex is imperfectly developed head pain is unusually rare.

Head pains are classified into Anæmic, Congestive, Toxic, Nervous, Sympathetic and Organic pains.

Anæmic headaches are typified in Chlorosis, and are due to impoverished blood. The pain is worse when the patient is up and less when reclining, consequently the patient sleeps well. It is worse in the afternoon and evening and better in the morning. Pressure on the carotids will bring it back. The blood pressure is below normal.

In the Congestive variety the symptoms are reversed in a way. Carotid pressure will cause it to disappear. The pain is worse while lying down and the sleep is poor. Such pain is found in plethoric persons or in drinkers. The blood pressure is above normal.

Toxic head pains are typified in nephritis, and may come from a hundred causes, from digestive disturbances to carbonic acid gas intoxication. The infectious diseases are ushered in by a toxic headache and the associated symptoms are of the greatest importance.

The Nervous headache is one of the vaguer forms and less easily recognized. It is well observed in cases of neurasthenia and especially in the headache of worry or of fright.

Sympathetic headaches are seen in cases of eye strain and in uterine troubles.

Lastly organic headaches are seen in meningitis and in neoplasms in the brain.

The location and character of the pain has, as a rule, but little dependence upon etiology. The location may be anywhere, and the pain may be dull, sharp, lancinating or may be only a vague feeling of soreness, and still belong to any one of the above classes. Attention is especially called to the necessity of a blood examination as well as an accurate measurement of the blood pressure, which the author takes up in detail. He recommends the Riva-

Rocca Sphygmomanometer and points out that the normal pressure is about 115 for women and 125 for men with this instrument. The urine is also of importance in ridding a patient of this variety of trouble, and should always be examined very carefully.

In the line of treatment Dr. Taylor begins by removing any irritating causes and then sets to work to build up the system in general, as it may be necessary. If necessary the quality of the blood is improved. Especially is the blood pressure of importance and he seeks to bring this to the normal by diet, tonic and even by vaso-depressors to a limited degree. Baths, electricity and good hygiene are stressed. For immediate relief he recommends ice caps, rest, laxatives, acetanelid in small doses, if one knows his patient and can watch him, as well as caffeine and numerous drugs as they are indicated, but he does not recommend morphia under any but the most strenuous circumstances. He says to first diagnose the case and then treat accordingly.

*Author's abstract.

